# Rural Populations and Health: Determinants, Disparities, and Solutions

**DOI:** 10.5888/pcd10.130097

**Published:** 2013-06-27

**Authors:** Laura Hall Downey

**Figure:**
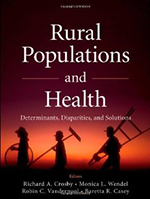


Health disparities are the “differences in [the] incidence, prevalence, morbidity, mortality and burden of diseases and other adverse health conditions that exist among specific population groups in the United States,” according to the National Institutes of Health (1). One of these population groups is rural Americans. Indeed, compared with their urban counterparts, rural communities have higher rates of preventable conditions such as obesity, diabetes, cancer, and injury, and higher rates of related high-risk health behaviors such as smoking, physical inactivity, poor diet, and limited use of seatbelts (2,3). A population 60 million strong, rural individuals are greatly influenced by geography, and so is their health. Whether through the physical terrain of their environment or the composition of their communities, including an aging population, lower socioeconomic status, and higher concentrations of ethnic and racial minorities, rural residents are at risk for negative health outcomes.

Despite growing emphasis on alleviating the health disparities that result from rural living, the existing literature used to teach and train public health professionals on rural health issues has focused on the health care system and access-to-care barriers among individuals. Little attention has been directed to rural public health, which includes population-based, preventive approaches to improving the physical, mental, and social well-being of rural residents. Because of the paucity of adequate textbooks for teaching rural public health, Drs Richard Crosby, Monica Wendel, Robin Vanderpool, and Baretta Casey collaborated with colleagues across the country who work in rural communities to share their expertise and innovative strategies for meeting the challenges of rural health disparities. The result is *Rural Populations and Health: Determinants, Disparities, and Solutions*. Many of the book’s chapter authors are members of the Centers for Disease Control and Prevention–funded Prevention Research Centers network (4).

The text is divided into 4 distinct parts: rural communities in context, rural public health systems, health partnerships in rural communities, and evidence-based practice in rural communities. Part 1 focuses on the history of rural public health and the definition of *rurality*. Part 2 provides an overview of rural public health systems, highlighting exemplars of rural public health practice from 4 states: Colorado, Kentucky, Alabama, and Iowa. Part 3 covers methods for identifying rural health disparities, conducting needs assessments, mobilizing coalitions, and building community capacity in rural communities. Part 4 explores in more depth evidence-based solutions to issues pertinent to rural health, including adolescent health, oral health, mental health, physical activity, prevention of farm-related injuries, cancer prevention and control, and tobacco-use prevention.

*Rural Populations and Health* fills a gap in the literature, offering a much-needed synthesis of information on rural communities from a distinct public health perspective. This timely and unique textbook has 3 primary strengths: its breadth of scope, its coherence (even as edited text), and its writing, which is easily accessible for a practice-focused audience. The breadth of topics discussed by the chapter authors is incredibly valuable — from confronting the complexity of defining rurality to addressing oral health in communities that lack the resources or infrastructure available to their urban/suburban counterparts and from engaging and mobilizing residents in improving health to addressing unmet mental health needs and food-related disparities. The rural context challenges many of our field’s best practices, because they are not easily translated into programs that work in these communities. Among its peers, this edited text is remarkably coherent across chapters, enabling the reader to make connections among different topics. Finally, the writing is practical and focused, not so dense that the application gets lost in the volume of words.

Overall, *Rural Populations and Health* content is applicable to professionals who work in various disciplines, including epidemiology, health promotion, public health services research, medicine, nursing, psychology, social work, government, and public policy. More specifically, *Rural Populations and Health* is a valuable teaching text that employs a much-needed population perspective.

The primary weakness of the book is that it covers some topics very well and misses other topics altogether. For example, the chapter on unintentional injury focuses solely on tractor rollovers. Injury caused by tractors is an important topic in some rural communities but likely not universal. Coverage of other unintentional injuries (eg, motor vehicle accidents, childhood injuries) and the challenges of rural communities in preventing and treating such injuries would improve the book. Although one book cannot possibly encompass all topics, this one did not provide in-depth coverage of several important rural health issues, such as alcohol and substance abuse, domestic violence, sexually transmitted infections, adolescent pregnancy, and diabetes prevention and management. This book, however, is the first of its kind; perhaps future editions will cover more of these important rural health issues.

*Rural Populations and Health* gives much-needed attention to public health practice among a group of people who persistently experience health disparities. Improved programming, a firmer evidence base, and dialogue prompted by this book among professionals can only move the health of rural communities forward, thereby contributing to the nation’s goals for *Healthy People 2020* (5).
